# The complete chloroplast genome of the endangered species *Triaenophora shennongjiaensis* (Orobanchaceae s.l.)

**DOI:** 10.1080/23802359.2018.1467242

**Published:** 2018-04-26

**Authors:** Zhi Xia, Jun Wen

**Affiliations:** a College of Agronomy, Henan Agricultural University, Zhengzhou, China;; b Department of Botany, National Museum of Natural History, Smithsonian Institution, Washington, DC, USA

**Keywords:** *Triaenophora shennongjiaensis*, chloroplast genome, illumina sequencing, Lamiales, Orobanchaceae sensu lato

## Abstract

*Triaenophora shennongjiaensis* (Orobanchaceae sensu lato) is a recently described rare and endangered species endemic to Central China. In this study, the complete chloroplast (cp) genome of *T. shennpongjiaensis* was assembled based on reads obtained with the Illumina HiSeq platform. The cp genome of *T. shennongjiaensis* was 15,5319 bp in length and contained a pair of inverted repeat (IR, 27,484 bp) regions separated by a small single copy (SSC, 15,450 bp) and a large single copy (LSC, 84,901 bp) region. It encoded 112 genes including 78 protein-coding genes, 30 tRNA genes, and eight ribosomal RNA genes. The overall AT content of *T. shennongjiaensis* cp genome is 61.9%. The maximum likelihood phylogenetic analysis supports *T. shennongjiaensis* as sister to *Rehmannia*. This result will be helpful for the systematics, conservation, and breeding programs of *Triaenophora*.

## Introduction

*Triaenophora* is a small endemic genus in Central China consisting of three narrowly distributed species formerly placed in Scrophulariaceae (Hong et al. 1998; Li et al. 2005). *Triaenophora shennongjiaensis* is an endangered species from the Shennongjia National Natural Reserve, Hubei, China (Li et al. 2005). Recent molecular systematic studies showed that *Triaenophora* is not part of Scrophulariaceae and have placed *Triaenophora* in Orobanchaceae s.l. (Albach et al. 2009; Xia et al. 2009). In this study, we report the complete chloroplast (cp) genome of *T. shennongjiaensis*.

The plant material of *T. shennongjiaensis* was sampled from Panlong Cavern, Shennongjia National Natural Reserve, Hubei, China. The voucher specimen (ZX-2017-0601) is kept at the Henan Agricultural University Herbarium (HEAC). Genomic DNA was extracted from leaf tissue using the Plant Genomic DNA Kit (DP305) from Tiangen Biotech (Beijing) Co., Ltd. (Beijing, China). DNA sample was randomly fragmented into 400–600 bp fragments using an ultrasonicator. An Illumina paired-end DNA library with 500-bp insert size was constructed using a NEBNext^®^ Ultra^TM^ DNA Library Prep Kit. Paired-end sequencing (2 × 150 bp) was conducted on an Illumina HiSeq × Ten platform.

The paired-end reads were qualitatively assessed and assembled with SPAdes 3.6.1 (Bankevich et al. 2012) using the *Rehmannia piasezkii* chloroplast genome sequence as a reference (GenBank accession KX636160) (Zeng et al. 2017). Small gaps in the assemblies were bridged with specific primers designed for PCR based on their flanking sequences and then by Sanger sequencing (Dong et al. 2013). Chloroplast genome annotation was performed with Plann (Huang and Cronk 2015). The cp genome sequence was submitted to GenBank (accession number MH071405).

**Figure 1.**
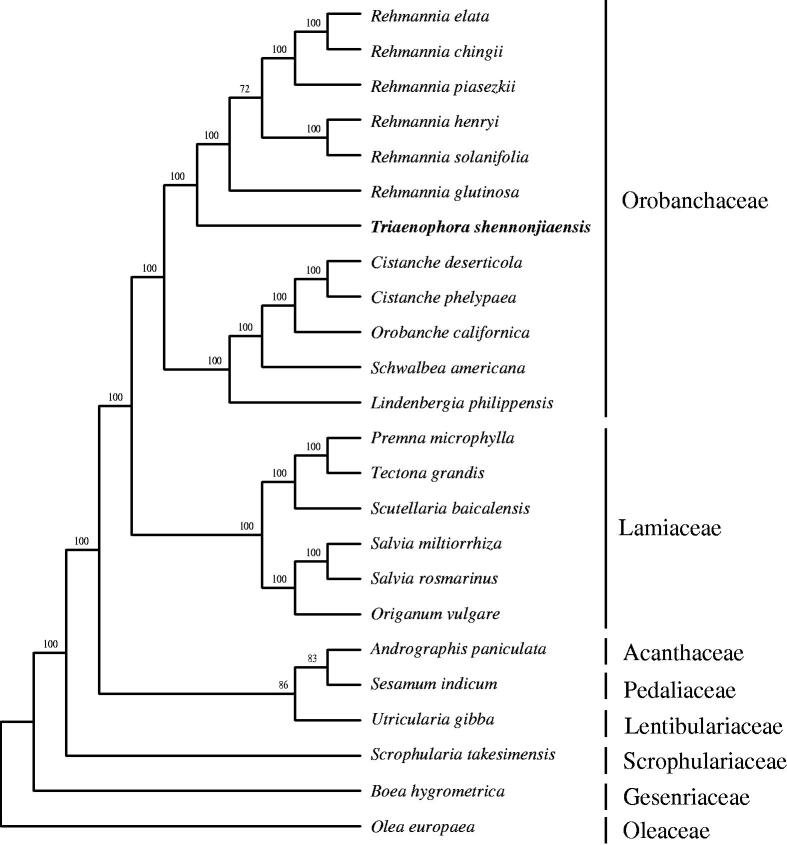
Maximum likelihood phylogenetic tree based on 24 complete chloroplast genome sequences. Accession numbers: *Cistanche deserticola* KC128846, *Cistanche phelypaea* HG515538, *Orobanche californica* HG515539, *Schwalbea americana* HG738866, *Lindenbergia philippensis* HG530133, *Rehmannia chingii* KX426347, *R. glutinosa* KX636157, *Rehmannia elata* KX636161, *Rehmannia piasezkii* KX636160, *Rehmannia solanifolia* KX636159, *Rehmannia henryi* KX636158, *Seasamum indicum* JN637766, *Scrophularia takesimensis* KM590983, *Premna microphylla* KM981744, *Tectona grandis* HF567869, *Scutellaria baicalensis* KR233163, *Origanum vulgare* JX880022, *Salvia miltiorrhiza* JX312195, *Salvia rosmarinus* KR232566, *Andrographis paniculate* KF150644, *Utricularia gibba* KC997777, *Boea hygrometrica* JN107811, *Olea europaea* GU931818 and *Triaenophora shennongjiaensis* MH071405. The number on each node indicates the bootstrap value.

The cp genome of *T. shennongjiaensis* was 155,319 bp in length and contains a pair of inverted repeat (IRa and IRb) regions of 27,484 bp, the large single copy (LSC) region and small single copy (SSC) region with the lengths of 84,901 and 15,450 bp, respectively (Figure 1). The whole chloroplast genome encoded 112 genes including 78 protein-coding genes (PCG), 30 tRNAs, and four rRNA operons. Among these genes, 15 genes (*atp*F, *ndh*A, *ndh*B, *pet*B, *pet*D, *rpl*16, *rpl*2, *rps*12, *rpl*16, *trn*A-UGC, *trn*G-GCC, *trn*I-GAU, *trn*K-UUU, *trn*L-UAA, and *trn*V-UAC) harboured one intron and two genes (*clp*P and *ycf*3) had two introns. Most genes occurred in a single copy, however, eight PCG genes (*ndh*B, *rpl*2, *rpl*23, *rps*7, *rps*12, *ycf*1, *ycf*2, and *ycf*15), seven tRNA genes (*trn*A-UGC, *trn*H-CAU, *trn*I-GAU, *trn*LCAA, *trn*N-GUU, *trn*R-ACG, and *trn*V-GAC), and four rRNA genes (rrn4.5, rrn5, rrn16, and rrn23) in the IR regions were duplicated. The overall AT content of the *T. shennongjiaensis* chloroplast genome is 61.9% and the corresponding values in LSC, SSC, and IR regions are 64.2, 68.1, and 57.5%, respectively.

Plastome sequences of 24 Lamiales species including *T. shennongjiaensis* were aligned with MAFFT (Katoh and Standley 2013). A maximum likelihood analysis was performed with the RAxML software (Stamatakis 2014) using 1000 bootstrap replicates. All sampled members of Orobanchaceae s.l. (*Lindenbergia*, *Rehmannia*, *Triaenophora*, *Cistanche*, *Orobanche*, and *Schwalbea*) formed a clade and *T. shennongjiaensis* was sister to *Rehmannia* (Figure 1). The chloroplast resource may be utilized for DNA barcoding, conservation genetics, and breeding of *Triaenophora*.
